# Evaluation of shallow foundation bearing capacity in the case of a two-layered soil and spatial variability in soil strength parameters

**DOI:** 10.1371/journal.pone.0231992

**Published:** 2020-04-29

**Authors:** Marcin Chwała, Wojciech Puła

**Affiliations:** Department of Geotechnology, Hydro Technology, and Underground and Hydro Engineering, Wroclaw University of Science and Technology, Wrocław, Poland; China University of Mining and Technology, CHINA

## Abstract

In this study, a probabilistic approach for evaluating the bearing capacity of surface footings is discussed. The evaluation is based on a kinematic approach. The considered substrate consists of two different layers of soil: a top layer formed of medium or dense sand followed by a layer of soft clay. The sand layer is assumed to be homogenous, whereas the undrained shear strength of the soft clay layer is assumed to be spatially variable, described by a lognormal random field. The random field is discretized according to Vanmarcke’s spatial averaging along dissipation regions in the considered failure mechanism. The mechanism utilizes plane strain conditions; however, due to consideration of the soil spatial variability in three dimensions, the impact of the length of the foundation on the random bearing capacity evaluation is considered in this study. As a result of the discretization procedure, a set of correlated random variables is obtained (each associated with an individual dissipation region in the failure mechanism). A series of numerical analyses are performed for two thicknesses of the first layer and a set of anisotropic correlation structures for the spatial variability of the undrained shear strength. The proposed method is computationally efficient and allows consideration of three-dimensional spatial variability in soil strength properties. The results are discussed and compared with those obtained by other methods.

## 1. Introduction

Bearing capacity evaluations of shallow foundations in the case of spatially variable soil are currently used primarily for two-dimensional analysis. One of the reasons for this situation is numerical efficiency, which limits the use of three-dimensional finite element formulations for random analyses. However, recent attempts have been made to solve such issues, e.g., [1]. Moreover, most existing probabilistic methods have been applied to single-layered soil. However, from an engineering perspective, two-layered soil is also a highly probable situation. An especially important situation is when the bottom layer is a soft soil and the top layer is a strong soil. This scenario has been extensively examined in the case of deterministic analyses (e.g., [2–5]); however, there have been very few studies on random analyses of two-layered soil (e.g., [6]). It is worth mentioning that it is possible to perform these types of two-dimensional analyses by using OptumCE software.

However, the lack of random approaches that are efficient and capable of introducing three-dimensional analyses was the motivation to propose an original approach that utilizes a kinematic approach in conjunction with Vanmarcke spatial averaging [7–9]. An analogous algorithm for plane strain conditions and a multiblock failure mechanism for a one-layered soil was proposed by Puła and Chwała [10]. The present study is also motivated by the engineering application to the problem of the bearing capacity of working platforms, which are usually constructed over soft subsoil to ensure safe traffic of heavy trucks. A working platform usually consists of compacted cohesionless material. Due to the controlled compaction procedure, the relatively homogenous soil layer is obtained. However, the spatial variability of the natural soil layer situated below the man-made layer can play a significant role in bearing capacity estimation. This situation is reflected in the numerical analyses. The importance of the problem can also be illustrated by investigating failures of working platforms, e.g., Channel Tunnel Rail Link Contract 310 [11] or Lublin (eastern Poland) road bypass (e.g., [12]).

In this study, as a deterministic background, a failure mechanism proposed by Michałowski and Shi [3] is assumed. The selected failure mechanism is dedicated for two-layered soil, i.e., the top layer is medium or dense sand, and the bottom layer is soft clay. According to the above described assumption, the spatial variability in soil strength is considered only in the bottom layer; thus, this approach characterizes the spatial variability in the undrained shear strength. The problem of designing working platforms is coded in BR 470 [13]. This study provides an efficient method for analysing such engineering problems and demonstrates that the random bearing capacity of two-layered soil in the case of spatial variability in the bottom layer can be efficiently evaluated by the developed kinematic approach. Moreover, despite the use of a two-dimensional mechanism, the spatial variability of the undrained shear strength in the soft clay layer is considered in three dimensions. As a result, an assessment of the impact of foundation length on random bearing capacity can be estimated. The importance of performing three-dimensional analyses is noted in this study. This is especially true if soil spatial variability is considered; thus, the spatial variability has a three-dimensional structure, and using two-dimensional simplifications can significantly influence the final results. A numerical algorithm is proposed in this study. According to this numerical procedure, numerous two-layered soil scenarios are analysed. Moreover, a simple approach to separate the impact of the thickness of the homogenous layer on random bearing capacity estimates from the impact of the spatial variability of the bottom layer and foundation length is provided in the study. The obtained results are shown and discussed in section 5.

In summary, the objective of this study was to propose an efficient approach for three-dimensional bearing capacity analysis of two-layered soil. The considered situation reflects the working platform scenario in which the top layer is a man-made layer of compacted cohesionless soil (in this study assumed to be homogenous) and the bottom layer is a natural, spatially variable, cohesive soil.

## 2. Algorithm

### 2.1. Failure mechanism description for random analyses

**Fig 1:**
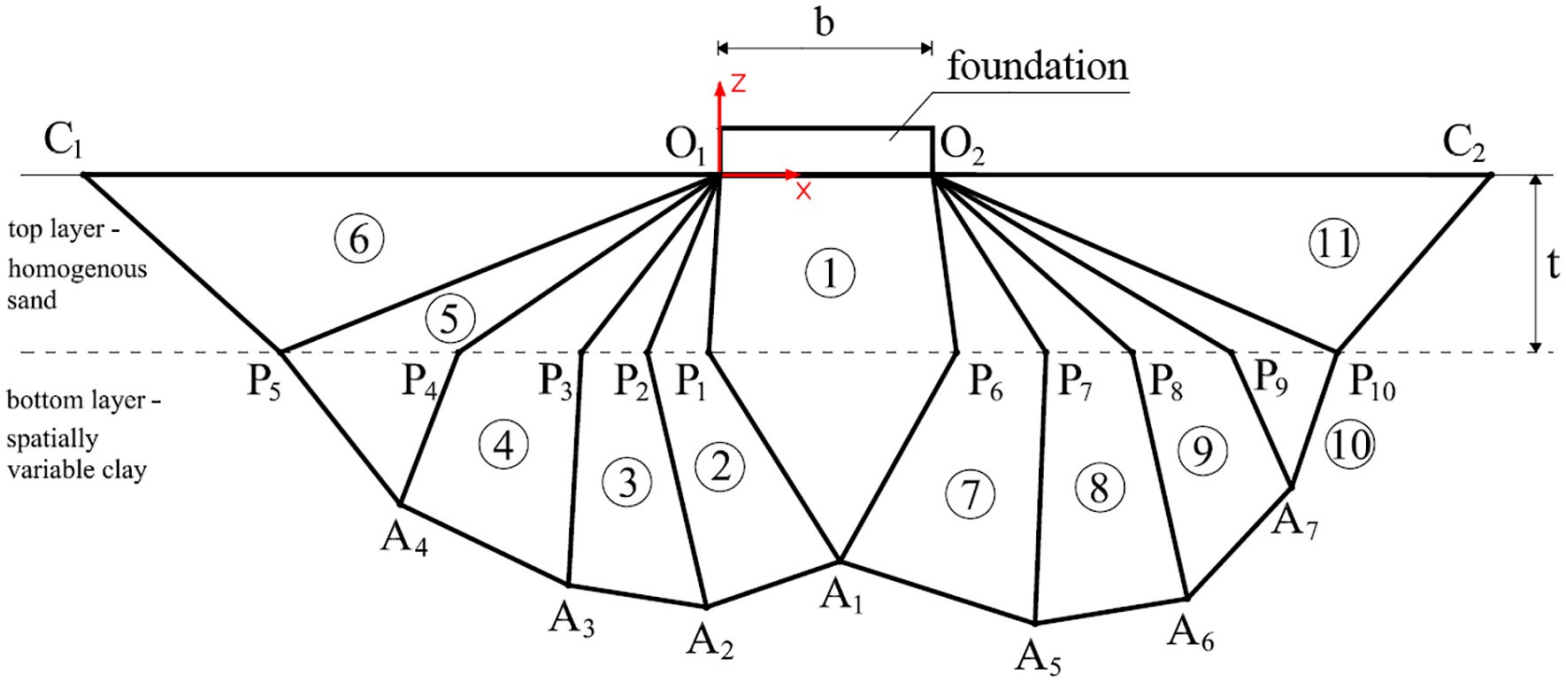
Rigid block numbering convention for the random version of the failure mechanism for two-layered soil. Note that *t* is the thickness of the top layer (homogenous sand) and that the bottom layer is assumed to be spatially variable clay.

The deterministic failure mechanism proposed by Michałowski and Shi [3] is used in this study. The geometry of the failure mechanism was modified to be suitable for random analyses. As a result, a new formula for bearing capacity is introduced. A detailed description of the random failure mechanism is provided below. The first assumption concerns the homogeneity of the top soil layer (note that in this study, a sand layer is considered to be the top layer). In the case of a working platform, the upper layer is practically a man-made material. Therefore, the spatial variability of this layer is neglected, and only a deterministic case is provided. As a result, the spatial variability in soil properties is considered for the bottom layer (purely cohesive soil). Moreover, for the bearing capacity estimations performed in this study, the bottom layer is assumed to be weightless. In the case of undrained conditions, the weight of the soil can be neglected. Moreover, as shown in Chwała [14] and also in the case of random analyses, the consideration of soil weight has a very limited impact on the final bearing capacity estimations. As in the deterministic version of the failure mechanism [3], the random failure mechanism proposed in this study consists of rigid blocks of soil that move with respect to each other. This failure mechanism is constructed in a particular manner so that there is no velocity discontinuity along the line that separates the top soil layer from the bottom layer. For the numerical analyses performed in this study, the failure mechanism of the 11th block is assumed. An example geometry and the rigid block numbering convention are shown in Fig 1.

**Fig 2:**
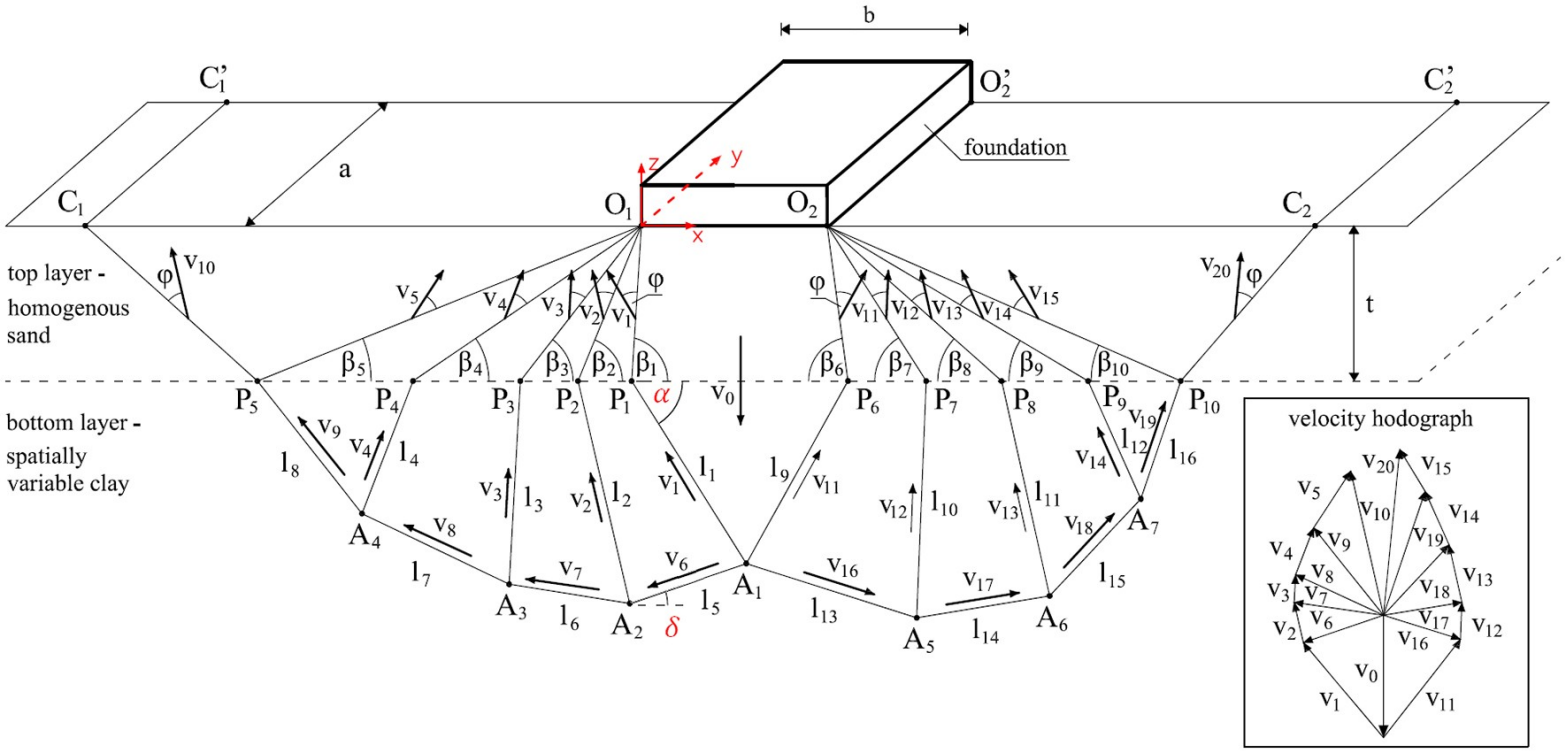
Detailed geometry of the failure mechanism. Note that if spatial variability of the bottom layer is assumed, the failure mechanism is non-symmetrical.

Note that the block shown in Fig 1 is a single rigid block, i.e., the block denoted by *O*_1_*P*_1_*A*_1_*A*_2_*P*_2_*O*_1_ defines a rigid block. Due to assumption of the homogeneity of the sand layer (top layer), all velocity jump vectors within this layer are inclined to the slip lines at the same angle that is equal to the assumed internal friction angle of sand *φ* (this angle is shown in Fig 2). Note that the velocities shown in Fig 2 can be obtained from the velocity hodograph shown in Fig 2. The velocity hodograph is constructed according to the geometrical relations between the velocity jump vectors shown in Fig 2.

The first noticeable difference when comparing the failure mechanism shown in Fig 2 with the deterministic failure mechanism is that in the random case, the failure geometry is no longer symmetric. Due to the different soil strength properties for each velocity discontinuity, the failure geometry is asymmetrical with respect to the vertical line passing through the foundation centre. An example random failure geometry is shown in detail in Fig 2. Based on Fig 2 and the upper bound theorem, the formula for bearing capacity can be derived. As an illustrative example, some parts of the bearing capacity formula are derived below. Based on the upper bound theorem, the upper limit of the bearing capacity can be found by comparison with the dissipation energy along velocity discontinuities and gravitational forces. Note that no overburden pressure is considered (a foundation is placed on the soil surface, as shown in Figs 1 and 2). According to results presented by Chwała [14], the self-weight of soil in the case of purely cohesive soil can be neglected when the spatial variability of soil strength is considered. As an example, the energy dissipated along the velocity discontinuity line P_1_A_1_ can be calculated as shown in Eq (1).

$$d_{P_{1}A_{1}}=c_{1}l_{1}v_{1}$$ (1)

where *c*_1_ and *l*_1_ are the cohesion and dissipation line length, respectively, and v_1_ is the velocity jump vector. All three values are shown in Fig 2. By using an analogous method, the remaining formulas for energy dissipation within the bottom layer can be derived. Moreover, because of the cohesionless character of the top layer, there is no energy dissipation within it (*c* = 0). Therefore, in the considered failure mechanism, the energy dissipates along sixteen lines within the bottom layer (see Fig 2). Another factor that influences the final bearing capacity estimation is the power of the gravitational forces on the top layer. The formula for region O_1_P_5_C_1_ is demonstrated in Eq (2).

$$g_{O_{1}P_{5}C_{1}}=\gamma F_{6}[v_{10}]$$ (2)

where *F*_6_ is the area of the rigid block indexed by 6 (see Fig 1). Note that in the case of block 5, the corresponding area *F*_5_ is the area of block 5 within the top layer. The velocity vector in brackets [v_10_] denotes the vertical component of the velocity jump vector v_10_. The unit weight of the sand layer is denoted by *γ*. By using an analogous method, the remaining formulas for the power of gravitational forces within the top layer can be derived. The power of the limit load that the foundation can be subjected to can be calculated by multiplying the bearing capacity *p* by the velocity v_0_ of the rigid block beneath the foundation (the foundation moves with the same velocity that is vertically oriented). Note that the formulas provided above assume by default that the calculations are performed for *a* = 1.0 m (see Fig 2); however, if other foundation lengths are considered, the corresponding value of *a* must be included. By summing all terms that relate to energy dissipation and gravitational forces and by transforming the resulting equation due to *p*, the following formula can be obtained:

$$p=\frac{1}{v_{0}}\left[\sum_{i=1}^{4}c_{i}v_{i}l_{i}+\sum_{i=5}^{8}c_{i}v_{i+1}l_{i}+\sum_{i=9}^{12}c_{i}v_{i+2}l_{i}+\sum_{i=13}^{16}c_{i}v_{i+3}l_{i}-\gamma \left(\sum_{j=1}^{5}F_{j+1}[v_{j+5}]+\sum_{j=11}^{15}F_{j-4}[v_{j+5}]+F_{1}\left[v_{0}\right]\right)\right]$$ (3)

where, as indicated earlier, v_*i*_ is the magnitude of the velocity jump vector along the slip line *i*, [v_*i*_] denotes the vertical component of velocity v_*i*_ (negative if upward), and *l*_*i*_ is the length of the slip surface *i*. In Eq (3), the undrained shear strengths are denoted simply by *c*_*i*_, where the index *i* is the same as that for the slip line lengths *l*_*i*_ (see Fig 2).

Design charts for calculating bearing capacity were provided in a paper by Michałowski and Shi [3]. As shown in Fig 2, when increasing the depth of the bottom layer (*t*), the critical value of *t* can be found. This critical value means that the failure mechanism is no longer deep (spread out in both soil layers) but becomes much shallower (spread out only in sand). This issue was described in detail by Michałowski and Shi [3], who showed that the critical depth of the weak layer depends on the angle of internal friction *φ* of the sand and the ratio *c*_*u*_/*γb*. Nevertheless, this issue is not considered in this study; thus, the examined scenarios relate to the case in which a deep failure mechanism occurs (as shown in Figs 1 or 2). The investigated situation is adequate for working platforms in which the top sand layer is relatively thin.

The formula shown in Eq (3) is crucial for determining the bearing capacity in the case of spatial variability in the strength properties of the bottom layer by applying different undrained shear strengths on each dissipation region. However, it is essential to properly calculate these undrained shear strengths. The method used in this study for that purpose is detailed in the next section. Moreover, Eq (3) provides the bearing capacity if the failure geometry is known (see Fig 2). Therefore, an optimization procedure is necessary to find the minimum value of the limit load because the method is based on the upper bound approach.

### 2.2. Spatial averaging

As indicated in the previous section, the method for determining average undrained shear strengths is a crucial element of the proposed approach. Because the sand layer is assumed to be non-random, the spatial averaging is subject only to the random field *X* that describes the spatial variability of the bottom layer. For this purpose, the method proposed by Puła and Chwała [15] is used here. Note that the approach described in the mentioned paper concerns a one-layered soil and Prandtl failure mechanism. However, with some modifications and adaptations, this approach can be utilized for the scenario considered in this study. The modified version of the spatial averaging procedure is described below. Additionally, to maintain the clarity of this paper, some general information is also provided below.

The approach is based on Vanmarcke’s spatial averaging concept [7–9]. Generally, the procedure replaces the need to generate a random field *X* by generating a set of correlated random variables (random vector ***X***_***V***_). Those random variables (*X*_*V*,1_, *X*_*V*,2_, …, *X*_*V*,*n*_) or random vector components correspond to the dissipation regions resulting from the considered failure mechanism. Due to the assumed stationarity of the random field, after the averaging procedure, the mean values of the random vector components are preserved; however, their variances are subject to reduction. The average is calculated as shown in Eq (4):

$$X_{V,i}=\frac{1}{\left|V_{i}\right|}\int \int \int X(x,y,z)dxdydz$$ (4)

where *V*_*i*_ is the averaging domain that corresponds to the dissipation region and *X* is the initial random field. Because spatial averaging is performed in three dimensions, the dissipation regions in this study are rectangles (see Fig 2). The variance of the new random variable *X*_*Vi*_ is given by:

$$Var\left(X_{V}\right)=\sigma_{V}^{2}=\gamma (V)\sigma_{X}^{2},$$ (5)

where $\sigma_{X}^{2}$ is the variance of the random field *X*. According to earlier experience (e.g., [16, 17]), in this study, the field *X* is assumed to be a lognormal random field. However, both isotropic and anisotropic correlation structures are examined. As a correlation function within the random field, the Gaussian covariance function was selected and used in the numerical analyses, as shown in Eq (6).

$$\rho (\Delta x,\Delta y,\Delta z)=exp\left\{-\left[{\left(\sqrt{\pi }\frac{\Delta x}{\theta_{x}}\right)}^{2}+{\left(\sqrt{\pi }\frac{\Delta y}{\theta_{y}}\right)}^{2}+{\left(\sqrt{\pi }\frac{\Delta z}{\theta_{z}}\right)}^{2}\right]\right\}$$ (6)

where *θ*_*x*_, *θ*_*y*_ and *θ*_*z*_ are fluctuation scales [7, 17]. To obtain the covariance function from Eq (5), the right-hand side of the equation must be multiplied by $\sigma_{X}^{2}$, i.e., $R\left(\Delta x,\Delta y,\Delta z\right)=\sigma_{X}^{2}\rho \left(\Delta x,\Delta y,\Delta z\right)$. Note that in this study, $\sigma_{X}^{2}=\sigma_{c_{u}}^{2}$. Eq (5) is applied to the three-dimensional scenario. Despite the assumed plane strain conditions, spatial averaging is performed in three dimensions. The coordinate system is shown in Fig 2. However, in further analyses, both horizontal fluctuation scales are assumed to be equal. Moreover, they are not distinguished and are denoted as *θ*_*h*_ (*θ*_*h*_ = *θ*_*x*_ = *θ*_*y*_). The fluctuation scale along the z direction is called the vertical fluctuation scale *θ*_*v*_ (*θ*_*v*_ = *θ*_*z*_). According to the failure mechanism shown in Fig 2, there are 16 regions in which spatial averaging is necessary. To determine these regions, a covariance matrix that describes the mutual correlations between each pair of regions is needed. To calculate the components of the covariance matrix, dedicated formulas must be derived. Derivation is possible due to the equations shown in Eq (7) (see [15, 18]).

$$Cov\left(X_{Vi},X_{Vj}\right)=\frac{1}{\left|V_{i}\right|\left|V_{j}\right|}\int_{V_{i}}^{}\int_{V_{j}}^{}R\left(x_{i},y_{i},z_{i},x_{j},y_{j},z_{j}\right)dV_{i}\left(x_{i},y_{i},z_{i}\right){dV}_{j}\left(x_{j},y_{j},z_{j}\right)$$ (7)

The above equation determines the covariance between two random variables *X*_*Vi*_ and *X*_*Vj*_ resulting from the averaging (Eq (4)) corresponding to the dissipation regions *V*_*i*_ and *V*_*j*_ (here, rectangles are shown in Fig 2; e.g., P_1_A_1_P’_1_A’_1_). Note that *i*, *j* = 1, …, 16. As a result, the final size of the covariance matrix is 16×16. The covariance matrix is symmetric and positively definite. Below, some formulas for the covariance matrix components are derived. First, consider the dissipation region P_1_A_1_P’_1_A’_1_ (points P’_1_ and A’_1_ are not shown in Fig 2, but their coordinates are the same as those of P_1_ and A_1_, respectively, except that the *y* coordinates are shifted by *α*). Fig 2 shows the angle *α* (coloured red), which is used for parametric representation of the region P_1_A_1_P’_1_A’_1_. Indeed, the considered region can be expressed by using the following parametrization (Eq (8)):

Eq.d, by using the following parametrization the considered region can be expresses to be derived. is needed.

$$x\left(t\right)=x_{P_{1}}+t\;\text{cos}\;\alpha$$ (8)

$$z\left(t\right)=z_{P_{1}}-t\;\text{sin}\;\alpha$$

By substituting Eqs (8) and (6) into Eq (7), the following formula for the new variance of region P_1_A_1_P’_1_A’_1_ is obtained:

$$Var\left(X_{P_{1}A_{1}},X_{P_{1}A_{1}}\right)=\frac{1}{{\left|P_{P_{1}A_{1}}\right|}^{2}}\int_{0}^{l_{1}}\int_{0}^{a}\int_{0}^{l_{1}}\int_{0}^{a}exp\left[-{\left(\sqrt{\pi }\frac{\left(y_{1}-y_{2}\right)}{\theta_{h}}\right)}^{2}\right]\times exp\left[-{\left(\sqrt{\pi }\frac{\left(t_{1}\;\text{cos}\;\alpha -t_{2}\;\text{cos}\;\alpha \right)}{\theta_{h}}\right)}^{2}\right]exp\left[-{\left(\sqrt{\pi }\frac{\left(t_{1}\;\text{sin}\;\alpha -t_{2}\;\text{sin}\;\alpha \right)}{\theta_{v}}\right)}^{2}\right]dy_{1}dt_{1}dy_{2}dt_{2}$$ (9)

Note that in Eq (9), the region P_1_A_1_P’_1_A’_1_ is denoted more simply as P_1_A_1_; this convention is also used later. Other variances of regions described by points P_i_ and A_i_ can be derived analogously.

In the case of region A_1_A_2_, the transformation shown in Eq (10) can be utilized as follows:

$$\begin{array}{c} x\left(t\right)=x_{A_{1}}+t\;\text{cos}\;\delta \\ z\left(t\right)=z_{A_{1}}-t\;\text{sin}\;\delta \end{array}$$ (10)

where the angle *δ* is shown in Fig 2 (coloured red).

$$Var\left(X_{A_{1}A_{2}},X_{A_{1}A_{2}}\right)=\frac{1}{{\left|P_{A_{1}A_{2}}\right|}^{2}}\int_{0}^{l_{5}}\int_{0}^{a}\int_{0}^{l_{5}}\int_{0}^{a}exp\left[-{\left(\sqrt{\pi }\frac{\left(y_{1}-y_{2}\right)}{\theta_{h}}\right)}^{2}\right]\times exp\left[-{\left(\sqrt{\pi }\frac{\left(t_{1}\;\text{cos}\;\alpha -t_{2}\;\text{cos}\;\alpha \right)}{\theta_{h}}\right)}^{2}\right]exp\left[-{\left(\sqrt{\pi }\frac{\left(t_{1}\;\text{sin}\;\alpha -t_{2}\;\text{sin}\;\alpha \right)}{\theta_{v}}\right)}^{2}\right]dy_{1}dt_{1}dy_{2}dt_{2}$$ (11)

Note that in Eqs (9) and (11), the coordinates along each axis are subtracted from each other. As a result, the coordinates of points P_1_ and A_1_ are reduced. However, the situation is different when the covariance between two different regions is calculated. To illustrate this situation, the formula for the covariance between regions P_1_A_1_ and A_1_A_2_ is given in Eq (12).

$$Cov\left(X_{P_{1}A_{1}},X_{A_{1}A_{2}}\right)=\frac{1}{\left|P_{P_{1}A_{1}}\right|\left|P_{A_{1}A_{2}}\right|}\int_{0}^{l_{1}}\int_{0}^{a}\int_{0}^{l_{5}}\int_{0}^{a}exp\left[-{\left(\sqrt{\pi }\frac{\left(z_{P_{1}}-t_{1}\;\text{sin}\;\alpha -z_{A_{1}}+t_{2}\;\text{sin}\;\delta \right)}{\theta_{h}}\right)}^{2}\right]\times exp\left[-{\left(\sqrt{\pi }\frac{\left(y_{1}-y_{2}\right)}{\theta_{h}}\right)}^{2}\right]exp\left[-{\left(\sqrt{\pi }\frac{\left(x_{P_{1}}+t_{1}\;\text{cos}\;\alpha -x_{A_{1}}-t_{2}\;\text{cos}\;\delta \right)}{\theta_{v}}\right)}^{2}\right]dy_{1}dt_{1}dy_{2}dt_{2}$$ (12)

Based on an analogous procedure, the other covariance matrix elements were derived. To enable presentation of the covariance matrix, a simplification in the naming convention is introduced. First, start from the components considered above, i.e., $w_{1}=Var\left(X_{P_{1}A_{1}},X_{P_{1}A_{1}}\right),w_{5}=Var\left(X_{A_{1}A_{2}},X_{A_{1}A_{2}}\right)$ and $k_{15}=Cov\left(X_{P_{1}A_{1}},X_{A_{1}A_{2}}\right)$. Namely, the variances are denoted by *w*_*i*_, where *i* is the index of the considered region (see Fig 2); the covariances are denoted by *k*_*ij*_, where *i* ≠ *j*. As a result, the covariance matrix is given in Eq (13).

$$C_{X}=\left[\begin{array}{ccc} \begin{array}{cc} \begin{array}{c} w_{1}\\ k_{1,2} \end{array} & \begin{array}{c} k_{1,2}\\ w_{2} \end{array} \end{array} & \begin{array}{cc} \begin{array}{c} k_{1,3}\\ k_{2,3} \end{array} & \begin{array}{c} \ldots \\ \ldots \end{array} \end{array} & \begin{array}{cc} \begin{array}{c} k_{1,15}\\ k_{1,15} \end{array} & \begin{array}{c} k_{1,16}\\ k_{1,16} \end{array} \end{array}\\ \begin{array}{cc} \begin{array}{c} k_{1,3}\\ \vdots \end{array} & \begin{array}{c} k_{2,3}\\ \vdots \end{array} \end{array} & \begin{array}{cc} \begin{array}{c} w_{3}\\ \vdots \end{array} & \begin{array}{c} \ldots \\ \ddots \end{array} \end{array} & \begin{array}{cc} \begin{array}{c} k_{1,15}\\ \vdots \end{array} & \begin{array}{c} k_{1,16}\\ \vdots \end{array} \end{array}\\ \begin{array}{cc} \begin{array}{c} k_{1,15}\\ k_{1,16} \end{array} & \begin{array}{c} k_{1,15}\\ k_{1,16} \end{array} \end{array} & \begin{array}{cc} \begin{array}{c} k_{1,15}\\ k_{1,16} \end{array} & \begin{array}{c} \ldots \\ \ldots \end{array} \end{array} & \begin{array}{cc} \begin{array}{c} w_{15}\\ k_{1,15} \end{array} & \begin{array}{c} k_{1,16}\\ w_{16} \end{array} \end{array} \end{array}\right]$$ (13)

In the presented method, the covariance matrix size depends on the number of dissipation regions resulting from the failure mechanism. The size given in Eq (13) is valid for the failure mechanism of the 11th block shown in Fig 2. However, if a more rigid block is considered, the size of the covariance matrix is correspondingly larger.

The method for determining the covariance matrix for the considered failure mechanism is provided above. Next, based on *C*_*X*_, the average set of undrained shear strengths is computed by the method described in Appendix A based on the approach proposed by Puła and Chwała [15]. Through the algorithm, the sixteen independent values of undrained shear strengths (*c*_*u*,1_, …, *C*_*u*_,_16_), each dedicated to a specific dissipation region, are transformed to the correlated values ($\overset{-}{c_{u,1}},\ldots ,\overset{-}{c_{u,16}}$). The transformation is based on the Cholesky decomposition of the covariance matrix (Eq (13)). More details on this issue are also provided in the numerical algorithm section.

### 2.3. Simulated annealing optimization scheme

**Fig 3:**
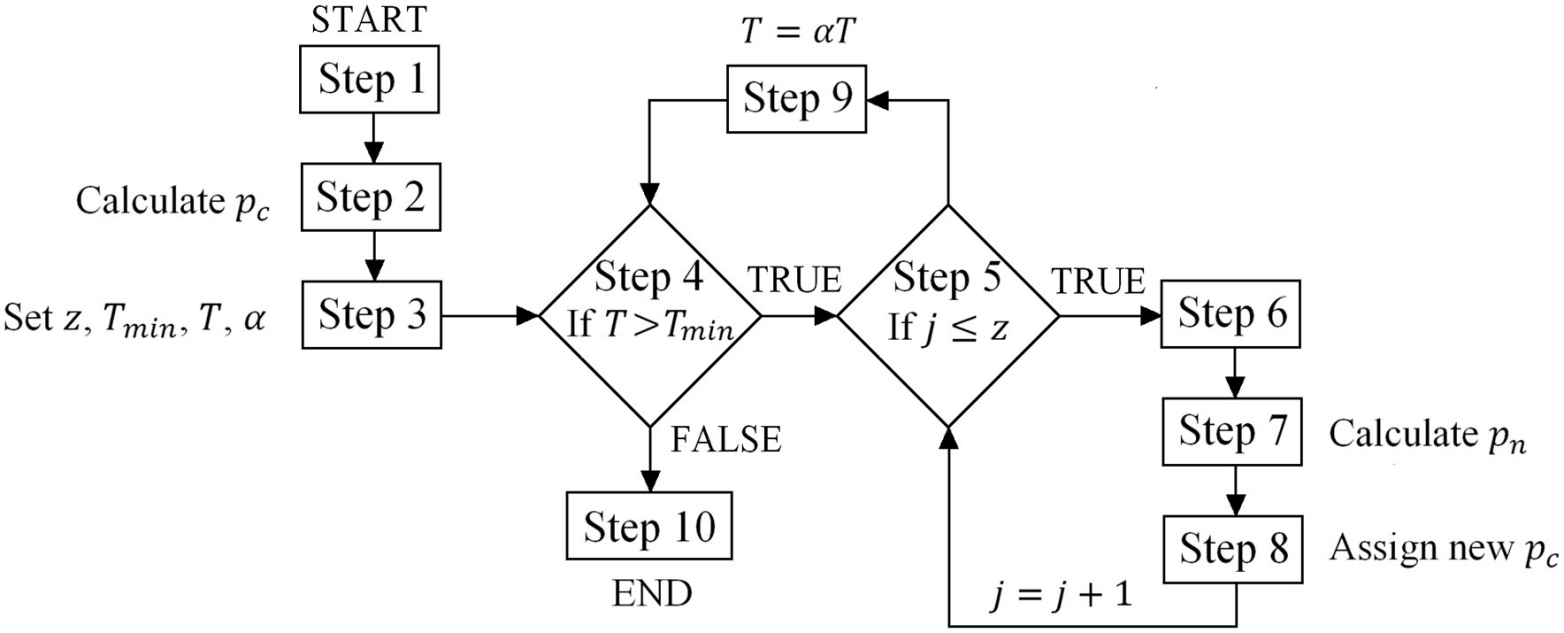
Flow chart for the optimization procedure. A detailed description is in the text.

As mentioned earlier, the procedure for calculating bearing capacity described in section 2.1 is applicable for a known failure geometry. However, before the covariance matrix is determined, the optimal failure geometry must be found. Here, optimal means the failure geometry that provides the lowest possible bearing capacity for the specified soil strength parameters. Therefore, the optimization procedure is crucial to use the multiblock failure mechanism shown in Fig 2. As a basic optimization procedure, a simulated annealing scheme is utilized in this study [19, 20]. Moreover, earlier authors’ experiences with the application of simulated annealing to optimize the geometry of kinematic failure mechanisms noted that this approach could be used here [10, 21]. As a result, the approach described below is created. The flow chart of the procedure is shown in Fig 3; to facilitate navigation, the step numbers have been added in the below description. Generally, the optimization method is based on slight changes in the failure geometry in subsequent simulations. The method starts with the initial geometry parameters (step 1) from which the first bearing capacity is calculated (step 2). After that, the control parameter values are set (step 3). For each simulation, the corresponding bearing capacity is calculated using Eq (3). One bearing capacity is called the current value *p*_*c*_, and this value is compared with those from the subsequent simulations *p*_*n*_. This method allows for accepting a worse solution than the current one (*p*_*c*_ < *p*_*n*_); however, the probability of such a scenario *P*_*a*_ decreases during the simulation process, and at the end of the simulation, this probability is nearly zero (only better solutions are accepted). Note that here, the better solution means the failure geometry that provides lower bearing capacity estimation. The concept of accepting worse solutions is due to overcoming local extrema; therefore, the final result is an approximation of the global extremum. In the simulated annealing scheme, there are some controlling parameters that control the simulation process (step 3). The formula used to calculate the acceptance probability is shown in Eq (14).

$$P_{a}=exp\left(\frac{p_{c}-p_{n}}{T}\right)$$ (14)

The parameter *T* is responsible for decreasing the acceptance probability *P*_*a*_ during the simulation process. Therefore, *T* also decreases during simulation. Note that Eq (14) is used if *p*_*c*_ < *p*_*n*_; however, if *p*_*n*_ < *p*_*c*_, the formula given in Eq (14) reaches a value greater than one, and as a result, a better solution is always accepted (step 8). According to the literature (e.g., [22]), to effectively set the optimization method, the average initial value of *P*_*a*_ should be approximately 0.5. The crucial element of the described optimization procedure is a procedure for generating a slightly different failure geometry based on the current one. This is a so-called neighbouring set of geometric parameters (step 6). This procedure is used to generate each failure geometry considered by the simulation process (omitting the first geometry that is determined arbitrarily). The geometry of the failure mechanism shown in Fig 2 has 18 degrees of freedom. Therefore, those 18 parameters are the arguments of the objective function that is intended to minimize Eq (3). As a result, the arguments of the procedure for determining a neighbouring failure geometry are those 18 parameters. The following parameters are selected for this purpose: $x_{C_{1}}$ and $x_{C_{2}}$ coordinates; $x_{P_{i}}$ coordinates, *i* = 1, …, 10; and lengths *l*_2_, *l*_3_, *l*_4_, *l*_10_, *l*_11_ and *l*_12_. Each geometric parameter is changed in a random order by increasing its initial value by an amount generated from a uniform distribution *U*[−0.01 m, 0.01 m]. Moreover, the procedure takes into account additional limitations on the values of the changed parameters, for example, to prevent overlapping of adjacent blocks, namely, to ensure that, e.g., $x_{P_{7}}>x_{P_{6}}$ (see Fig 2). As mentioned earlier, for a new set of geometric parameters, the bearing capacity *p*_*n*_ is calculated using Eq (3) (step 7) and compared with the current value *p*_*c*_ (step 8). The acceptance probability of a worse solution is modified by the parameter *T*; however, some simulations are performed under constant *P*_*a*_. In this study, for each *P*_*a*_ level, *z* = 2000 simulations are carried out (step 5). Then, *P*_*a*_ is decreased by modifying the value of *T*, namely, by multiplying *T* by an additional parameter: *αT* (step 9). The parameter *α* is kept constant during the simulation process and based on the literature and earlier experiences is assumed to be equal to 0.9. The simulation process continues as long as *T* > *T*_*min*_ (step 4). In this study, *T*_*min*_ = 10^−8^. When *T* reaches *T*_*min*_, the algorithm ends, and the current bearing capacity *p*_*c*_ is treated as the optimal bearing capacity (step 10). Note that the values of the parameters that control the simulation process have to be selected individually for the considered issue.

**Fig 4:**
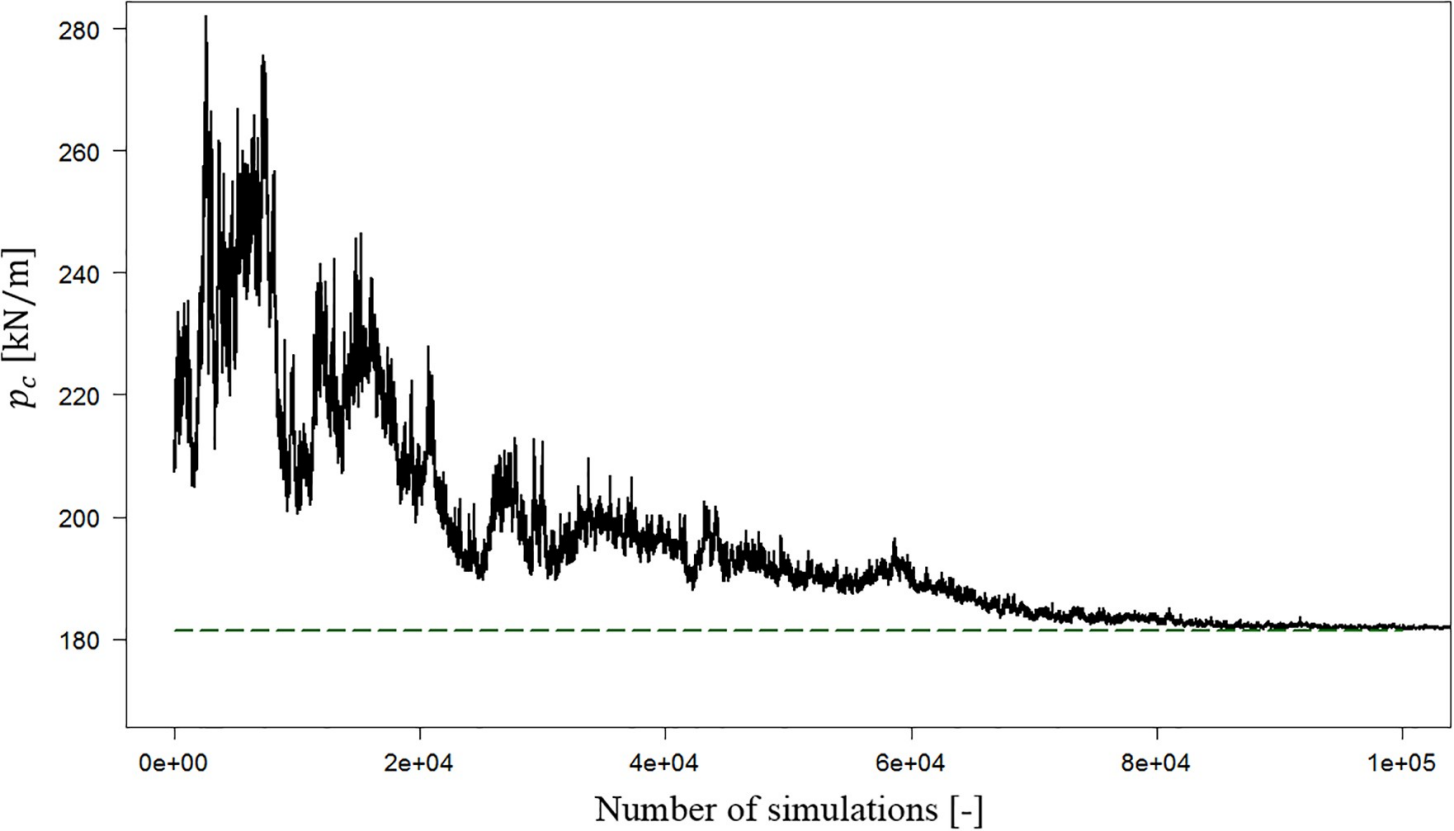
Value of the current bearing capacity *p*_*c*_ as a function of the number of simulations in the simulated annealing procedure. The horizontal dashed line shows the final bearing capacity that is equal to that obtained by Michałowski and Shi [3]. The example was calculated for the following parameters to make the comparison possible: *b* = 1.0 *m*, *γ* = 20 *kN*/*m*^3^, *c*_*u*_*/γb* = 1.0, *t* = 10 *m* and *φ* = 35°.

According to the performed tests, the parameters chosen for the purpose of this study are definitely appropriate in terms of the achieved accuracy. The geometry of the failure mechanism obtained by using the optimization procedure described above for a non-random case was identical to the geometry obtained by Michałowski and Shi [3]. One example of this comparison is shown in Fig 4. For the initial geometry, the bearing capacity is approximately 210 kN/m. However, at the beginning stage of the simulation process, the value reaches over 280 kN/m. Despite this, during a simulation process with decreasing *P*_*a*_, the current bearing capacity *p*_*c*_ moves towards the optimal value.

The results obtained for the optimization procedure were also compared with the results obtained using finite element limit analysis implemented in OptumG2 [23] software for different numbers of finite elements and calculation methods. The obtained results are juxtaposed in Table 1. The results were obtained under plane strain conditions. To enable their comparison to the three-dimensional case, additional deterministic analyses were carried out by using ZSoil software [24]. In the case of *t* = 0.5 *m* bearing capacities of 142.5 kN/m, 140.6 kN/m and 136.8 kN/m were obtained for foundation lengths 6 m, 10 m and 16 m, respectively. In the case of *t* = 1.0 *m* bearing capacities of 215.6 kN/m, 198.7 kN/m and 191.2 kN/m were obtained for foundation lengths 6 m, 10 m and 16 m, respectively. As expected, the obtained bearing capacities are greater than those obtained for the plane strain condition and tends to decrease with an increase of foundation length.

**Table 1:** Comparison of bearing capacity estimations for *b* = 1.0 *m*, *γ* 18 *kN*/*m*^3^, *φ* = 35° and two thicknesses of the sand layer: *t* = 0.5 *m* and *t* = 1.0 *m*.

Finite limit analysis calculation parameters		*t* = 0.5 m			*t* = 1.0 m		
Computation method	No. of finite elements	Finite limit analyses [kN/m]	This study [kN/m]	Difference [%]	Finite limit analyses [kN/m]	This study [kN/m]	Difference [%]
Standard	1000	149.518	124.35	+20.2	194.487	174.53	+11.4
	10000	123.227		-0.9	168.636		-3.4
Mesh adaptivity	1000	117.526		-5.5	168.528		-3.4
	10000	110.590		-11.1	157.371		-9.8

### 2.4. Algorithm summary

In sections 2.1, 2.2 and 2.3, all the necessary components to build the numerical procedure are described. The connection of those elements is provided in the next section to find the probabilistic characteristic of the random bearing capacity. As a governing framework of the numerical procedure, a Monte Carlo method is used. Moreover, the flow chart of the algorithm layout is provided in the next section.

## 3. Numerical procedure

**Fig 5:**
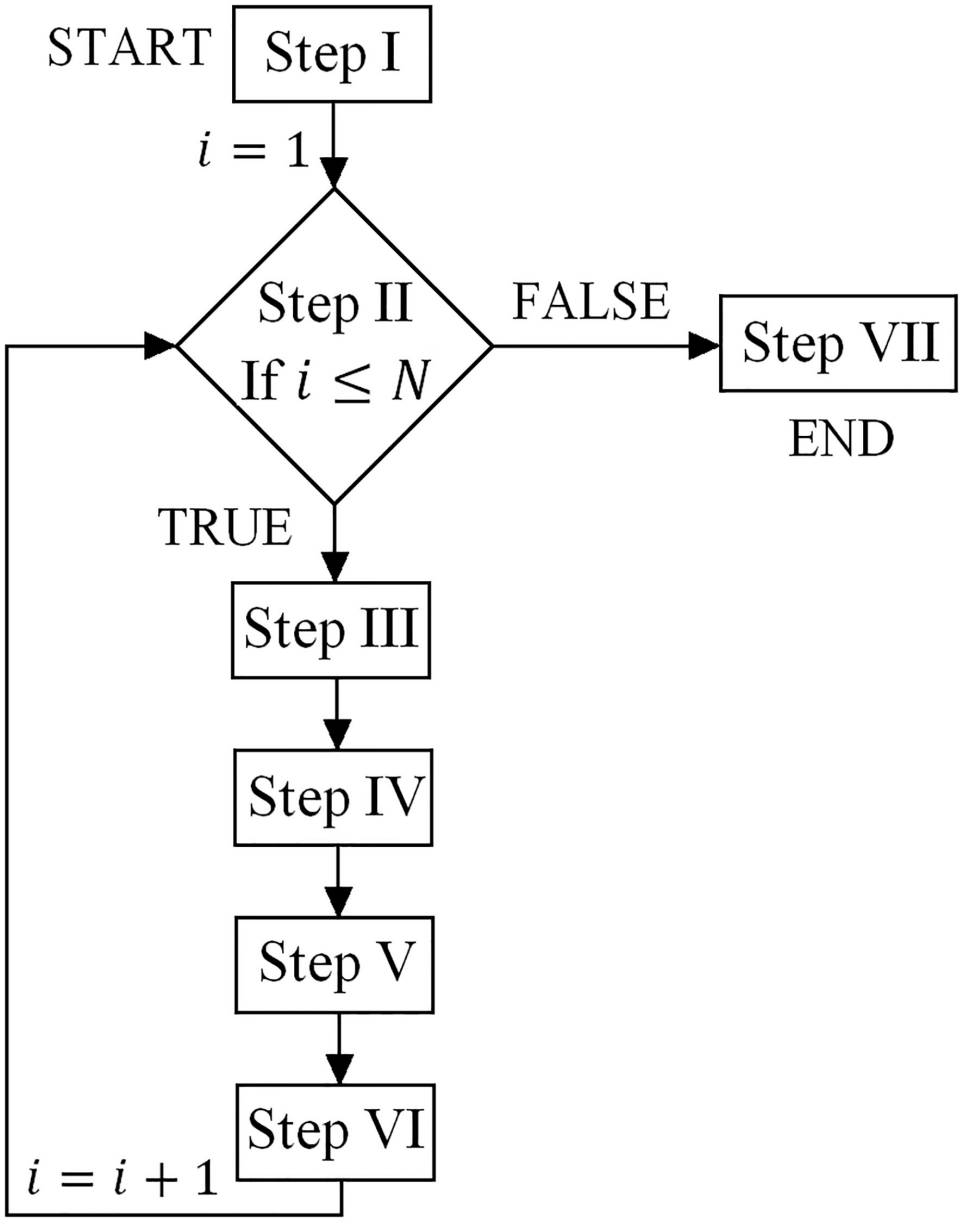
Flow chart of the numerical procedure. A detailed description is provided in the text.

The numerical procedure for a one Monte Carlo simulation is provided below (see also Fig 5):

Step I introduces the values of the initial parameters. Set *i* = 1.
Step II starts Monte Carlo loop. If *i* ≤ *N* follow the steps below. If *i* > *N* go to step VII.
Step III generates 16 independent values of undrained shear strengths via a pseudo-random number generator $Y_{c_{u}}=(c_{Y,u,1},\ldots ,c_{Y,u,16}$). All values are generated from the underlying normal distribution ($\mu_{Y,c_{u}}$ and $\sigma_{{Y,c}_{u}}$) for the initial stationary lognormal random field ($\mu_{c_{u}}$ and $\sigma_{c_{u}}$).
Step IV finds the optimal bearing capacity based on the soil parameters from step III transformed to the lognormal distribution $X_{c_{u}}=(c_{X,u,1},\ldots ,c_{X,u,16})$ by using the optimization procedure described in section 2.3.
Step V determines the correlated undrained shear strengths by transforming the independent values of undrained shear strength obtained in step III to correlated values using the covariance matrix (Eq (13)) determined for the optimal failure geometry from step IV. For this purpose, the algorithm shown in Appendix A is used. This algorithm is based on the Cholesky decomposition (e.g., [25]) of the covariance matrix *C*_*X*_. Generally, the correlated parameters are obtained by calculating the product of a vector of uncorrelated parameters obtained in step III that was standardized and a triangular matrix that is the result of the Cholesky decomposition of *C*_*X*_. Finally, a set of correlated values for the undrained shear strength is obtained ($\overset{-}{c_{u,1}},\ldots ,\overset{-}{c_{u,16}}$). These values are correlated by the covariance matrix *C*_*X*_.
Step VI calculates the final bearing capacity by using the correlated undrained shear strengths ($\overset{-}{c_{u,1}},\ldots ,\overset{-}{c_{u,16}}$) obtained in Step V. For this purpose, the optimization procedure from section 2.3 is used again. The resulting failure geometry and the corresponding bearing capacity are returned as the final results of the algorithm. Set *i* = *i* + 1, and go to step II.
Step VII provides the set of *N* bearing capacity estimates.

In this study, for all performed analyses, the number of Monte Carlo simulations *N* is equal to 1000. The proposed approach is computationally efficient; namely, one three-dimensional simulation takes approximately 1 minute for one core of a standard notebook.

**Fig 6:**
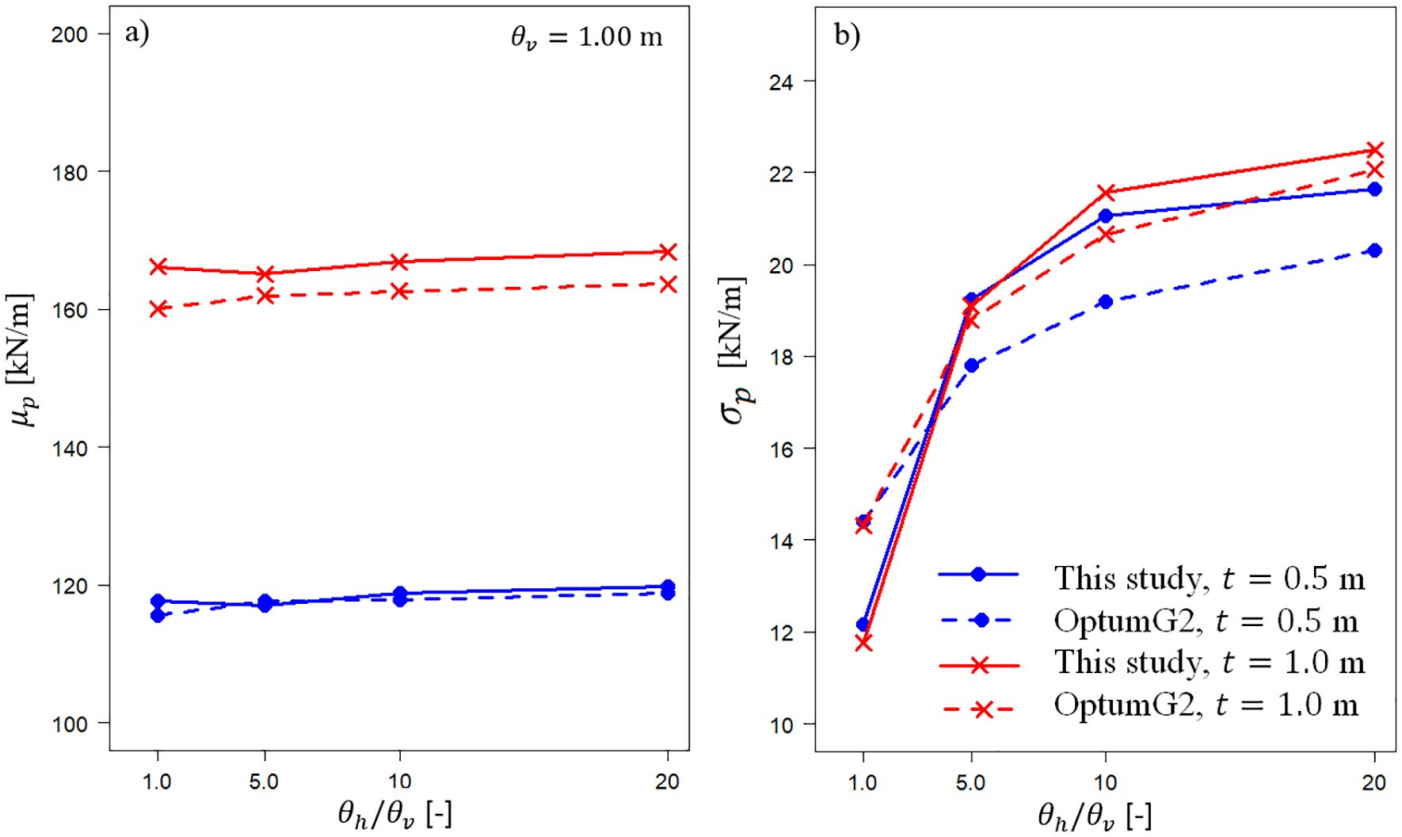
Comparison of the results obtained with the algorithm developed in this study and those obtained with OptumG2 software. Two scenarios were considered for *t* = 0.5 m and *t* = 1.0 m. (a) mean values of bearing capacity; (b) standard deviations of bearing capacity.

The numerical procedure detailed above allows consideration of three-dimensional soil spatial variability in the bearing capacity estimations for two-layered soil. Unfortunately, there are no published results that can be compared with the approach proposed in this study. However, this is possible in the case of plane strain conditions. In this particular case, the random finite limit analysis can be used for a comparison by using OptumG2 software. The comparison is made for $\mu_{c_{u}}=20\;kPa$ and $v_{c_{u}}=0.25$ for two thicknesses of the top layer: *t* = 0.5 m and *t* = 1.0 m. The results for *θ*_*v*_ = 1.0 *m* plotted as a function of *θ*_*h*_/*θ*_*v*_ are shown in Fig 6. The observed differences between both approaches are acceptable. Moreover, to some extent, the differences can be explained by the different correlation function assumed in the analyses performed by OptumG2 software (the Markovian one).

## 4. Considered scenarios

The main objective of this study is to assess the impact of a homogenous sand layer (top layer) on the overall random bearing capacity estimation in the case of a two-layered soil. A spatially variable, purely cohesive soil was assumed as the bottom layer. Moreover, the considered scenarios were selected to ensure creation of the failure mechanism shown in Fig 2. Therefore, relatively small thicknesses of the sand layer were examined. These thicknesses are suitable for working platform problems. The impact of vertical and horizontal fluctuation scales on random bearing capacity was investigated. Three vertical fluctuation scales were selected for numerical analyses, namely, *θ*_*v*_ = 0.25 m, *θ*_*v*_ = 0.50 m and *θ*_*v*_ = 1.00 m. These values were recently indicated by many authors, e.g., [26]. In the case of horizontal fluctuation scales, the values of *θ*_*h*_ = *θ*_*v*_, *θ*_*h*_ = 5*θ*_*v*_, *θ*_*h*_ = 10*θ*_*v*_ and *θ*_*h*_ = 20*θ*_*v*_ were examined. The two-dimensional failure mechanism was extended to three dimensions (Fig 2) to enable consideration of three-dimensional spatial averaging. As a result, the length of the averaging domain *a* was also examined. The following lengths were considered in the numerical analyses: *a* = 1.0 m, *a* = 3.0 m, *a* = 6.0 m and *a* = 10.0 m. The analyses that considered the impact of fluctuation scales were performed for two values of the undrained shear strength coefficient of variation: $v_{c_{u}}=0.25$ and $v_{c_{u}}=0.50$. The mean value of undrained shear strength was assumed to be $\mu_{c_{u}}=20\;\text{kPa}$. Numerical analyses of all of the abovementioned parameter combinations were performed for two thicknesses of the top layer, i.e., *t* = 0.5 m and *t* = 1.0 m. The information for all scenarios that considered the impact of fluctuation scales is juxtaposed in Table 2.

**Table 2:** Combinations of fluctuation scales and averaging domain length a used in the numerical analyses. Note that the shown scenarios are repeated for two thicknesses of the sand layer and two values of $v_{c_{u}}$, as detailed in the text. Therefore, 192 scenarios were analysed.

*a*	*θ*_*v*_/*θ*_*h*_	*θ*_*v*_/*θ*_*h*_	*θ*_*v*_/*θ*_*h*_	*θ*_*v*_/*θ*_*h*_
*a* = 1.0 m	0.25 m/0.25 m	0.25 m/1.25 m	0.25 m/2.5 m	0.25 m/5.0 m
*a* = 3.0 m	0.25 m/0.25 m	0.25 m/1.25 m	0.25 m/2.5 m	0.25 m/5.0 m
*a* = 6.0 m	0.25 m/0.25 m	0.25 m/1.25 m	0.25 m/2.5 m	0.25 m/5.0 m
*a* = 10.0 m	0.25 m/0.25 m	0.25 m/1.25 m	0.25 m/2.5 m	0.25 m/5.0 m
*a*	*θ*_*v*_/*θ*_*h*_	*θ*_*v*_/*θ*_*h*_	*θ*_*v*_/*θ*_*h*_	*θ*_*v*_/*θ*_*h*_
*a* = 1.0 m	0.5 m/0.5 m	0.5 m/2.5 m	0.5 m/5.0 m	0.5 m/10.0 m
*a* = 3.0 m	0.5 m/0.5 m	0.5 m/2.5 m	0.5 m/5.0 m	0.5 m/10.0 m
*a* = 6.0 m	0.5 m/0.5 m	0.5 m/2.5 m	0.5 m/5.0 m	0.5 m/10.0 m
*a* = 10.0 m	0.5 m/0.5 m	0.5 m/2.5 m	0.5 m/5.0 m	0.5 m/10.0 m
*a*	*θ*_*v*_/*θ*_*h*_	*θ*_*v*_/*θ*_*h*_	*θ*_*v*_/*θ*_*h*_	*θ*_*v*_/*θ*_*h*_
*a* = 1.0 m	1.0 m/1.0 m	1.0 m/5.0 m	1.0 m/10.0 m	1.0 m/20.0 m
*a* = 3.0 m	1.0 m/1.0 m	1.0 m/5.0 m	1.0 m/10.0 m	1.0 m/20.0 m
*a* = 6.0 m	1.0 m/1.0 m	1.0 m/5.0 m	1.0 m/10.0 m	1.0 m/20.0 m
*a* = 10.0 m	1.0 m/1.0 m	1.0 m/5.0 m	1.0 m/10.0 m	1.0 m/20.0 m

Moreover, for comparison purposes, scenarios with assumed infinite horizontal and vertical fluctuation scales were examined. By assuming *θ*_*v*_ = *θ*_*h*_ = ∞, a simple situation where the undrained shear strength is described by a single random variable is obtained. In such a case, there is no spatial averaging; therefore, the obtained standard deviations or coefficients of variation of bearing capacity are conservative estimates of the true values. These scenarios were intended to be analysed to separate the influence of dumping the spatial variability impact on the final bearing capacity coefficient of variation for the deterministic top layer of sand.

## 5. Results

**Fig 7:**
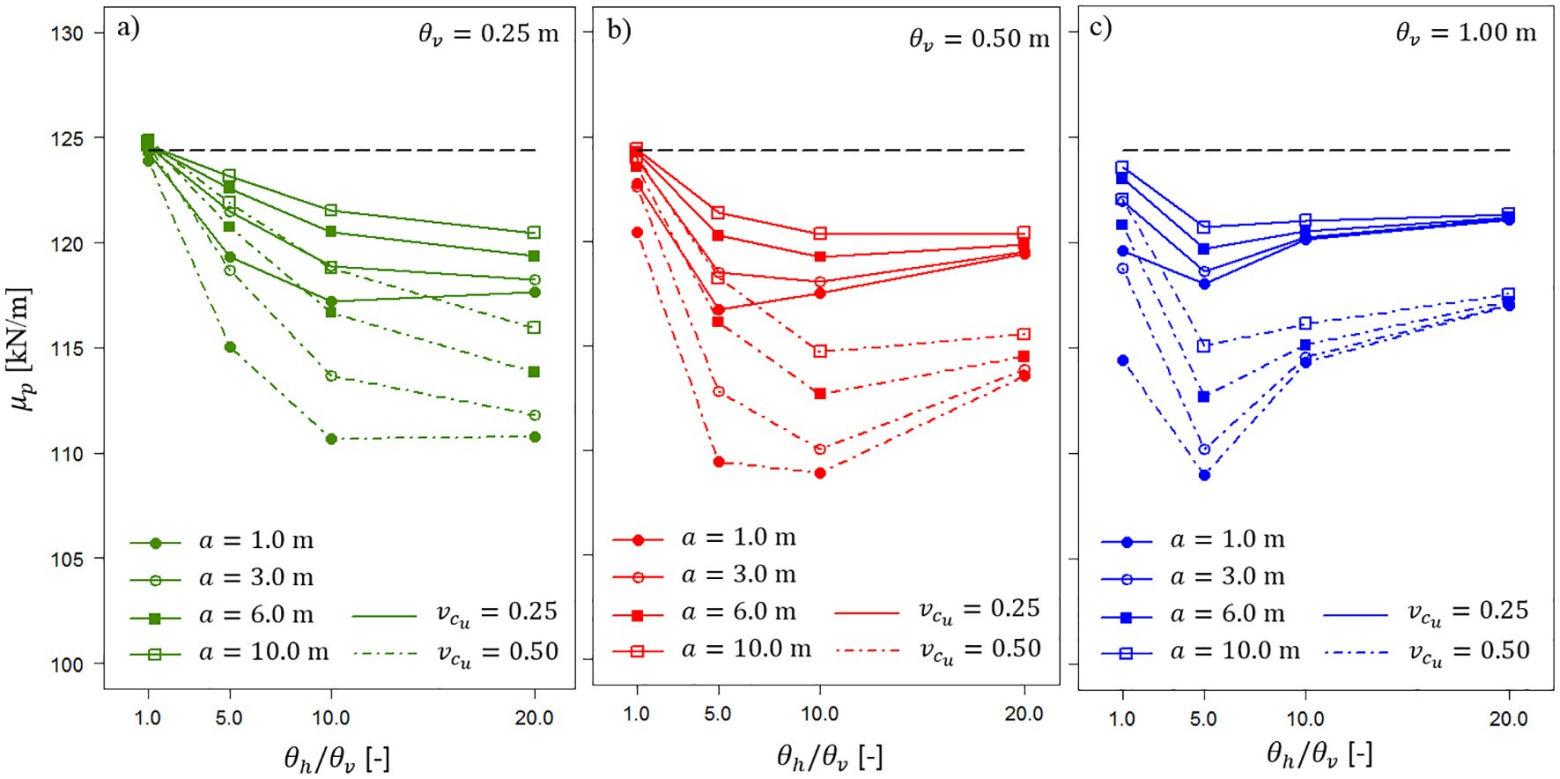
Bearing capacity mean values *μ*_*p*_ for *t* = 0.5 m as a function of the horizontal fluctuation scale and length of the averaging domain *a*. Three vertical fluctuation scales are considered. The dashed lines represent $v_{c_{u}}=0.5$, and the solid lines represent $v_{c_{u}}=0.25$.

Based on the Monte Carlo analyses, the mean values of the resulting bearing capacity *μ*_*p*_ and corresponding bearing capacity coefficients of variation were obtained. In Fig 7, the bearing capacity mean values obtained for *t* = 0.5 m are shown. The dashed lines represent $v_{c_{u}}=0.5$, and the solid lines represent $v_{c_{u}}=0.25$. Lower *μ*_*p*_ values are observed for greater $v_{c_{u}}$ values. This effect can be a result of using a lognormal probability distribution of *c*_*u*_; a similar effect was described in [27]. In Fig 7a, 7b and 7c, the results for *θ*_*v*_ = 0.25 m, *θ*_*v*_ = 0.5 m and *θ*_*v*_ = 1.0 m are provided, respectively. Some interesting observations can be made based on Fig 7. First, *μ*_*p*_, shown as a function of the horizontal fluctuation scale, reaches a minimum value for *θ*_*v*_ = 0.5 m and *θ*_*v*_ = 1.0 m. In the case of *θ*_*v*_ = 0.5 m, the minimum value in most of the considered cases is observed for *θ*_*v*_/*θ*_*h*_ = 10. However, for *θ*_*v*_ = 1.0 m, the minimum value is observed for *θ*_*v*_/*θ*_*h*_ = 5. By multiplying the vertical fluctuation scale by the ratio for which the minimum is observed in both cases, the value of the horizontal fluctuation scale, *θ*_*v*_ = 5.0 m, is obtained. This observation indicates that the observed worst-case correlation length (e.g., [28]) for the mean value is independent of the vertical fluctuation scale. Note that Fig 7a does not refute this observation.

**Fig 8:**
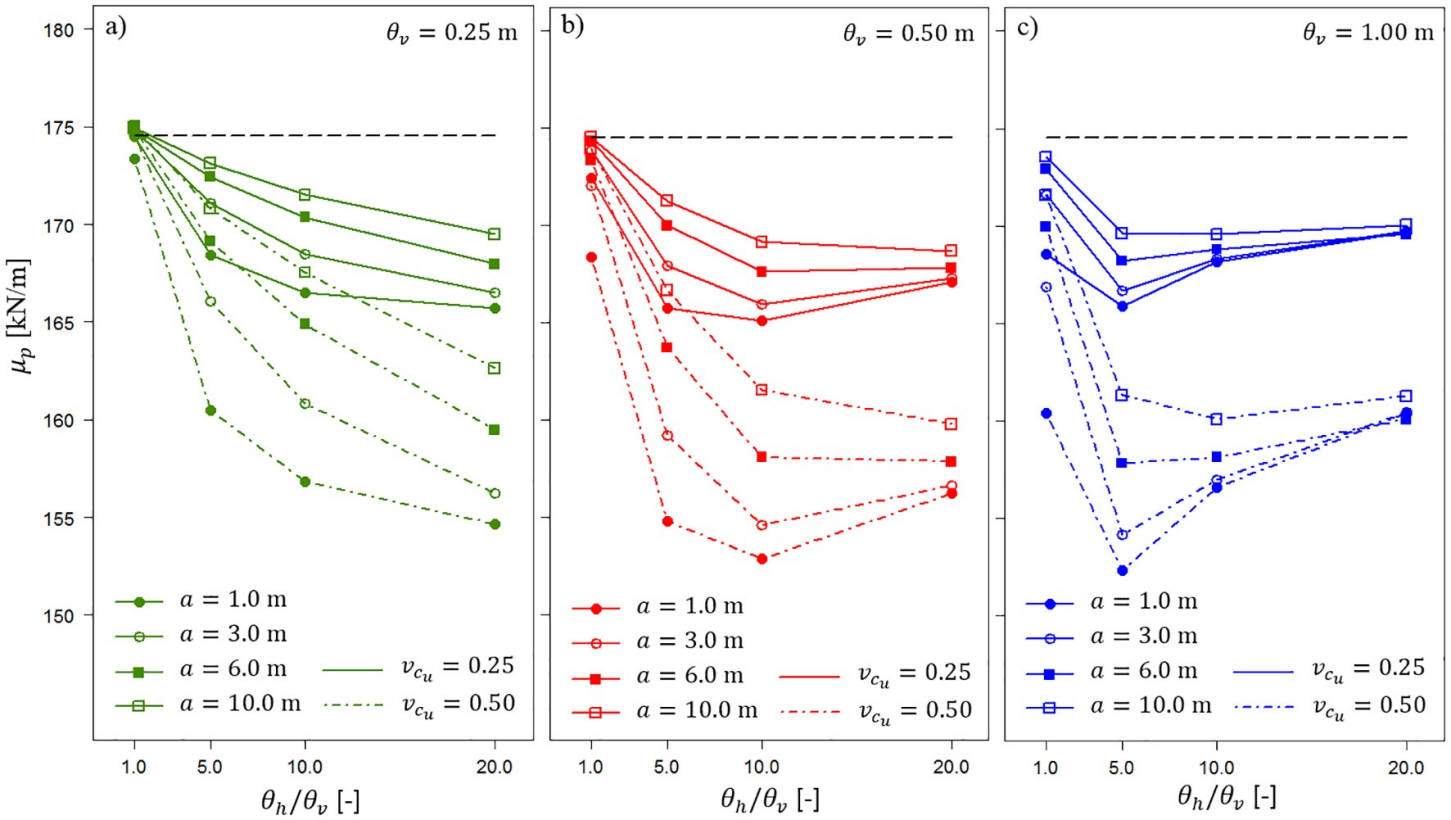
Bearing capacity mean values *μ*_*p*_ for *t* = 1.0 m as a function of the horizontal fluctuation scale and length of the averaging domain *a*. Three vertical fluctuation scales are considered.

Fig 7 clearly shows the differences in the bearing capacity mean values for different values of *a*. In each case, a longer averaging domain provides a higher *μ*_*p*_. This can also be explained by the abovementioned effect of obtaining a lower *μ*_*p*_ for a greater coefficient of variation *c*_*u*_. Thus, a longer averaging domain *a* provides greater averaging; therefore, the overall coefficient of variation *c*_*u*_ decreases as the variances defined in Eq (5) are more significantly reduced. It is worth mentioning that all obtained bearing capacity mean values are below the values of the deterministic scenario that is denoted by the horizontal dashed line. Note that the relative differences in the obtained *μ*_*p*_ values are not very significant (see values on the vertical axis). Analogous observations can be made for *t* = 1.0 m (Fig 8). However, the relative differences in the obtained *μ*_*p*_ values are smaller than those for *t* = 0.5 m. This demonstrates the effect of damping the impact of soil spatial variability on bearing capacity with increasing thickness of the homogenous layer *t*. This effect is described later in more detail.

**Fig 9:**
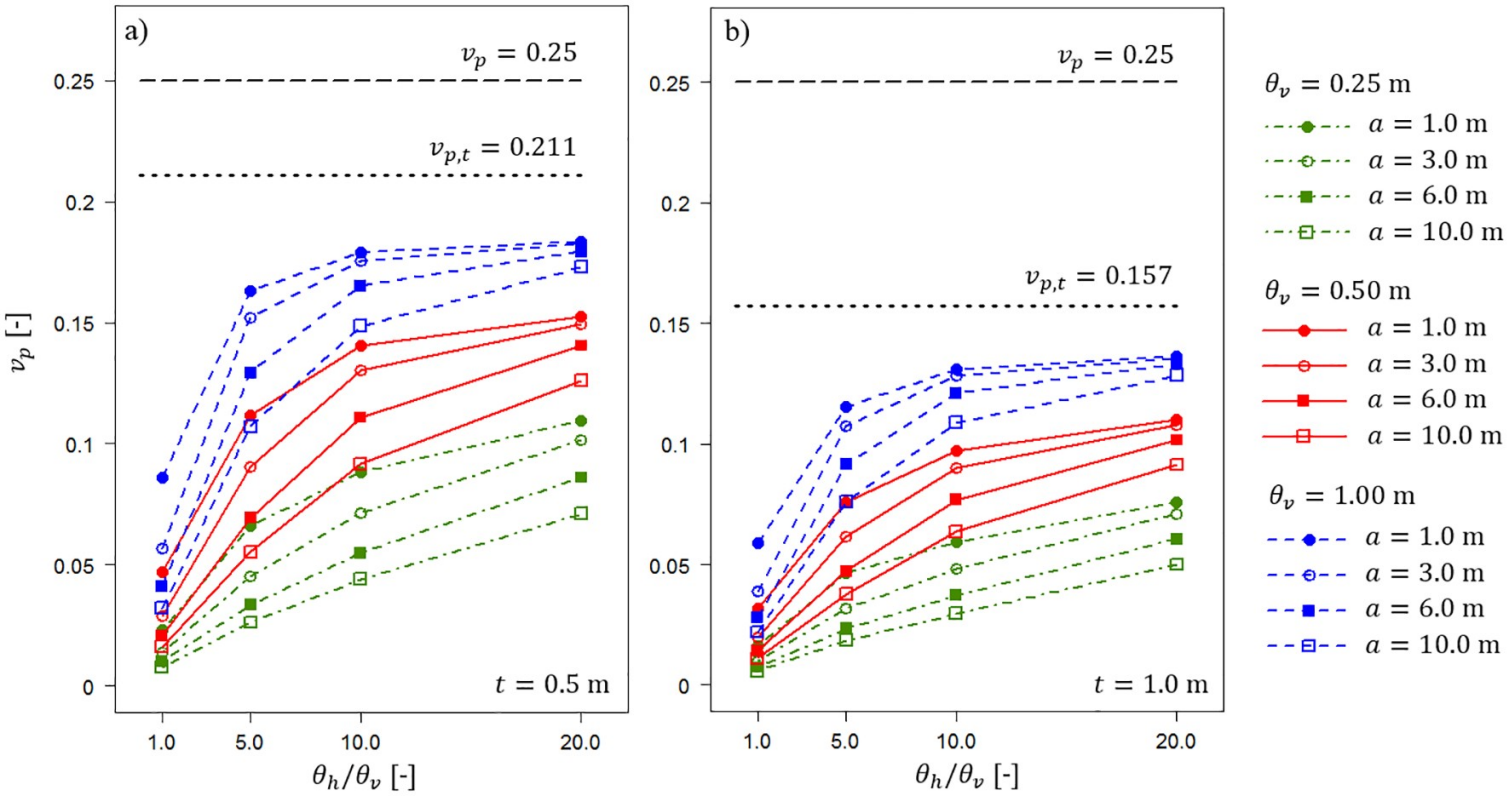
Bearing capacity coefficient of variation *v*_*p*_ for $v_{c_{u}}=0.25$ as a function of the horizontal fluctuation scale and length of the averaging domain *a*. Three vertical fluctuation scales and two thicknesses of the sand layer, i.e., t = 0.5 m (a) and t = 1.0 m (b), are considered. Detailed descriptions are provided in the text.

Very significant differences among the considered scenarios are observed in the coefficient of variation of bearing capacity *v*_*p*_. The results for $v_{c_{u}}=0.25$ are shown in Fig 9. The horizontal dashed and dotted lines shown in Fig 6 indicate *v*_*p*_ for special scenarios. The dashed line indicates the coefficient of variation of bearing capacity for *t* = 0 and infinite values of the vertical and horizontal fluctuation scales. In this case, the considered issue is degenerated into a one-layered purely cohesive soil with no spatial variability and $v_{c_{u}}=0.25$. Therefore, the resulting coefficient of variation of bearing capacity $v_{p}=v_{c_{u}}=0.25$ (as shown in Fig 9). However, the dotted lines in Fig 9a and 9b indicate the resulting coefficient of variation of bearing capacity for the value of *t* specified in each figure and infinite values of the vertical and horizontal fluctuation scales. For *t* = 0.5 m, the corresponding *v*_*p*,*t*_ = 0.211; thus, the coefficient of variation of bearing capacity decreases in comparison with the scenario in which *t* = 0 m. A further decrease is observed for *t* = 1.0 m, for which *v*_*p*,*t*_ 0.157. Thus, it is easy to understand why the *v*_*p*_ values observed for scenarios with finite fluctuation scales (see Table 1) are lower than *v*_*p*,*t*_. As shown in Fig 9, a shorter vertical fluctuation scale provides a smaller value of *v*_*p*_. The dotted lines represent the limit values of *v*_*p*_ when the fluctuation scales reach infinity. The information is intentionally presented in the form shown in Fig 9 to separate the impact of the homogenous sand layer from the impact of fluctuation scales on the resulting bearing capacity coefficients of variation. As expected, for a longer averaging domain *a*, a lower *v*_*p*_ is obtained. The observed differences between the extreme values, i.e., *a* = 1 m and *a* = 10 m, are significant, and they indicate the necessity of considering the real dimension of the structure if soil spatial variability is taken into account. In such situations, the two-dimensional simplification of three-dimensional issues provides more conservative bearing capacity estimates. This conclusion has recently been noted [1,21,29].

**Fig 10:**
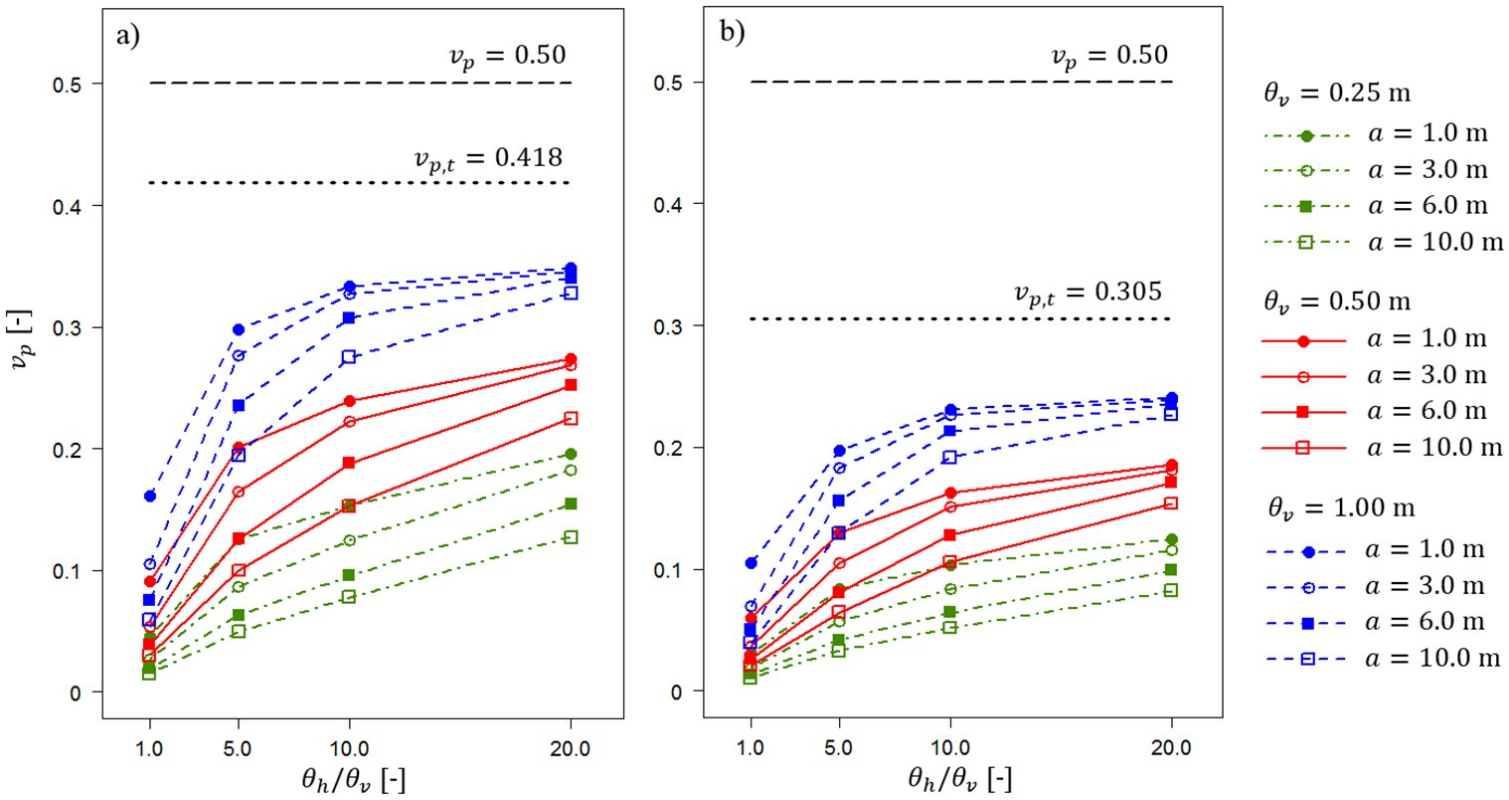
Bearing capacity coefficient of variation *v*_*p*_ for $v_{c_{u}}=0.5$ as a function of the horizontal fluctuation scale and length of the averaging domain *a*. Three vertical fluctuation scales and two thicknesses of the sand layer, i.e., *t* = 0.5 m and *t* = 1.0 m, are considered. Detailed descriptions are provided in the text.

Results analogous to those shown in Fig 9 for $v_{c_{u}}=0.5$ are shown in Fig 10. The horizontal dashed and dotted lines have the same meaning as those in Fig 9.

**Fig 11:**
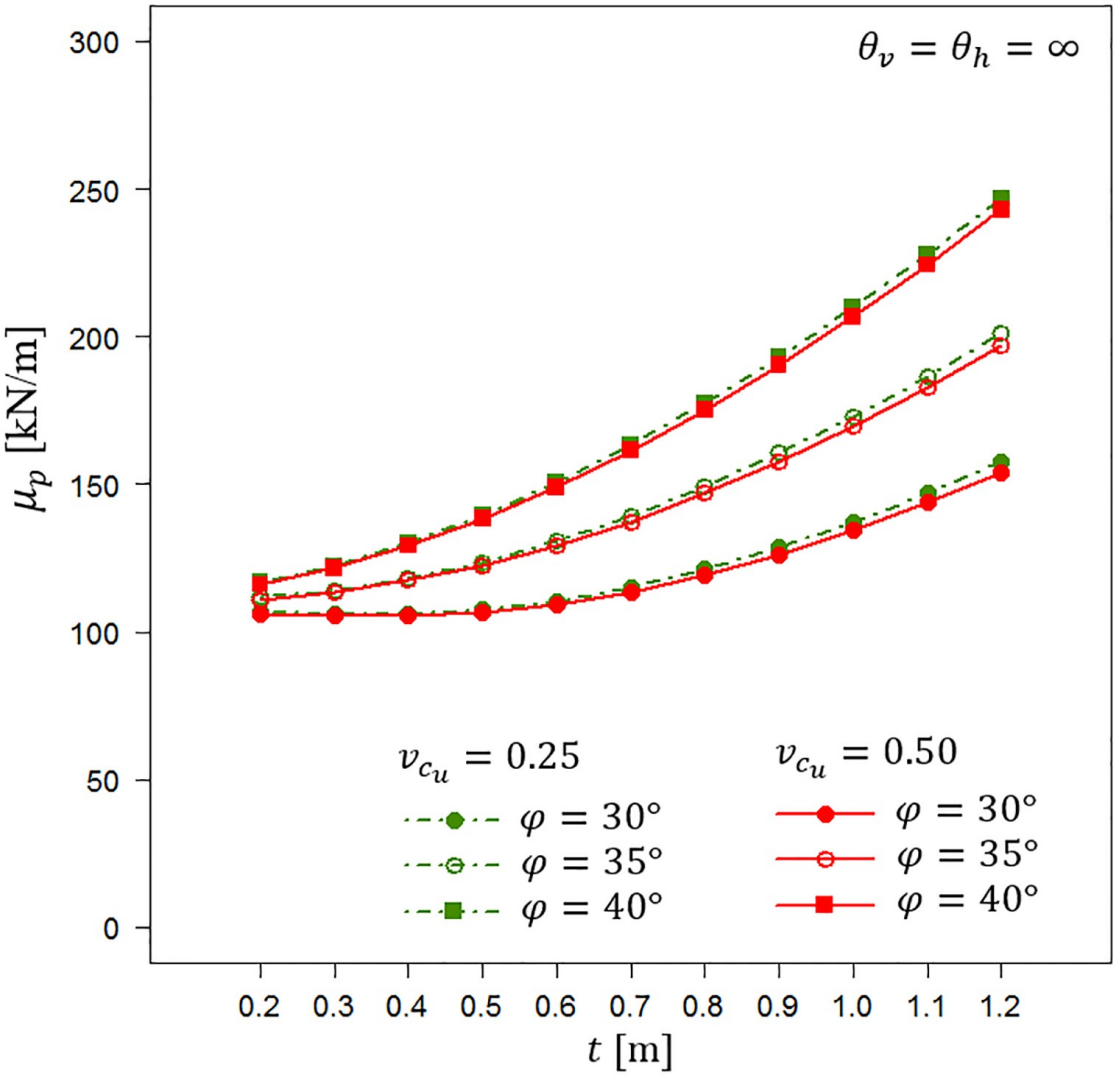
Bearing capacity mean values *μ*_*p*_ for $v_{c_{u}}=0.25$ and $v_{c_{u}}=0.5$ as a function of the sand layer thickness *t*. Three values of the angle of internal friction are considered.

**Fig 12:**
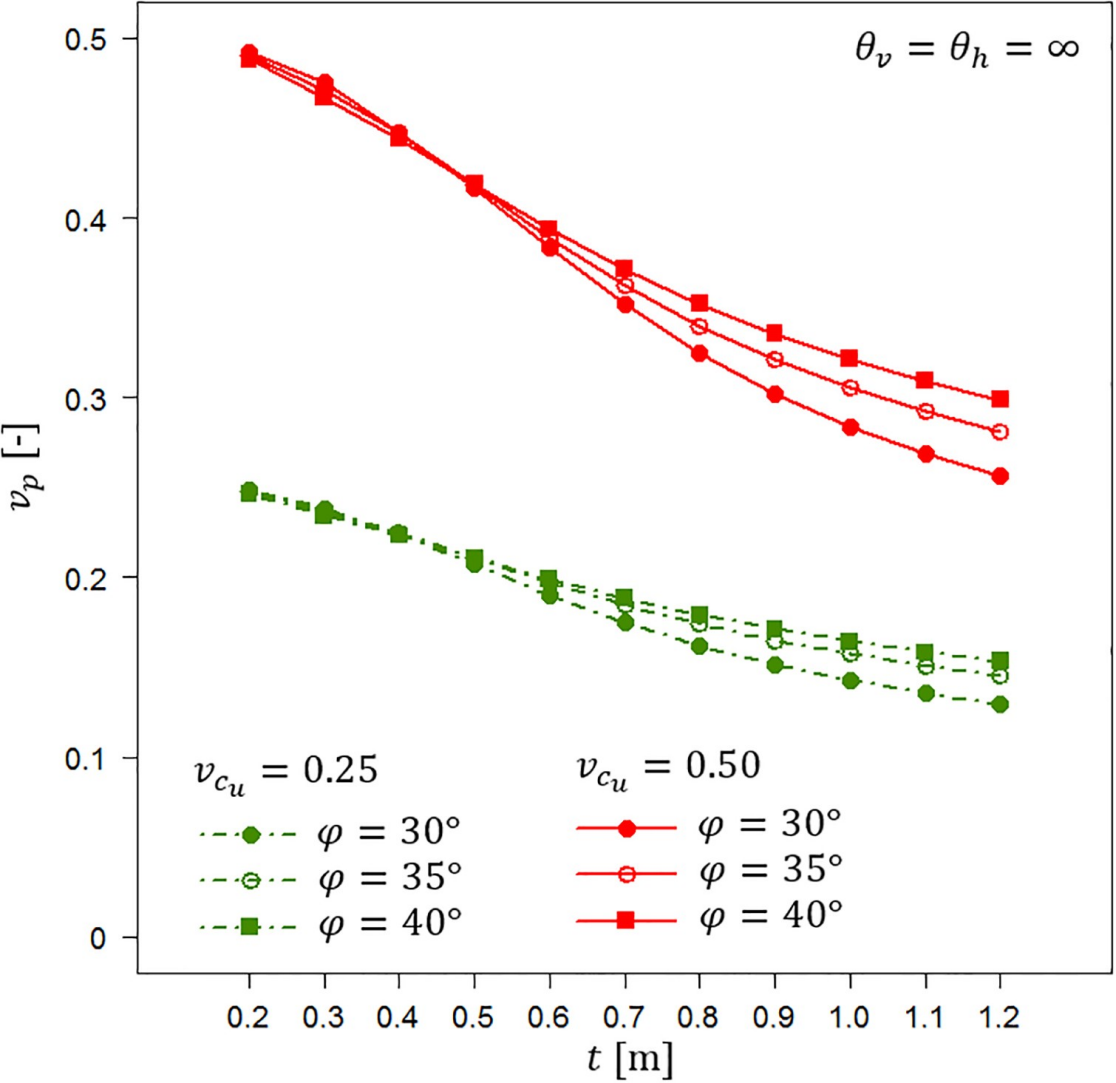
Bearing capacity coefficient of variation *v*_*p*_ for $v_{c_{u}}=0.25$ and $v_{c_{u}}=0.5$ as a function of the sand layer thickness *t*. Three values of the angle of internal friction are considered.

Both Figs 9 and 10 indicate that calculating *v*_*p*,*t*_ can be a very easy method to conservatively estimate the random bearing capacity in the case of a lack of information about fluctuation scales. The reduction in the bearing capacity coefficient of variation is a result of considering only the homogenous soil layer. Therefore, it can easily be estimated. To illustrate that possibility, Figs 11 and 12 show the results obtained by analysing scenarios with *θ*_*v*_ = *θ*_*h*_ = ∞. First, in Fig 11, the obtained bearing capacity mean values are plotted as a function of the homogenous sand layer thickness *t*. Fig 11 presents the results for $v_{c_{u}}=0.25$ and $v_{c_{u}}=0.50$. For each undrained shear strength coefficient of variation, three values of internal angle are considered, i.e., *φ* = 30°, *φ* = 35° and *φ* = 40°. Generally, the bearing capacity mean values increase with an increase in the sand layer thickness. Moreover, greater *μ*_*p*_ values are observed for greater angles of internal friction that characterize the sand layer. The differences between the scenarios with $v_{c_{u}}=0.25$ and $v_{c_{u}}=0.50$ are practically negligible.

Fig 12 shows the bearing capacity coefficients of variation that correspond to the mean values shown in Fig 11. The obtained results indicate that *v*_*p*_ decreases with an increase in the sand layer thickness. Moreover, the observed differences in *v*_*p*_ between different friction angles are relatively narrow. A simplified method for which the results are shown in Figs 11 and 12 can be used to estimate the reduction factor in the bearing capacity coefficient of variation that results from the thickness of the homogenous sand layer.

## 6. Summary and conclusions

An original adaptation of the two-dimensional failure mechanism for two-layered soil proposed by Michałowski and Shi [3] to random analyses is shown in the present study. The adaptation was necessary for numerical analyses that are based on the upper bound approach and are dedicated to the scenario of a spatially variable bottom layer. A method of Vanmarcke’s spatial averaging in conjunction with a kinematic failure mechanism [15] was used in this study. The use of this method requires its adaptation to the considered scenario. As a result, an original approach for random analysis of the bearing capacity of two-layered soil and three-dimensional spatial averaging was proposed in this study. The created algorithm is capable of considering all important parameters, such as geometrical parameters, thickness of the top layer, its unit density and fluctuation scales, that describe the variability of undrained shear strength along the vertical and horizontal directions. The proposed approach is very efficient (one three-dimensional simulation takes approximately 1 minute for one core of a standard notebook). The method is dedicated to a situation in which the top layer is homogenous sand (generally cohesionless soil) and the bottom layer is purely cohesive soil that is spatially variable. This arrangement of the soil layers has practical meaning because it reflects the conditions in which working platforms operate, where the top layer is man-made, and the assumption that it is homogenous is justified. The proposed approach is in accordance with the upper bound theorem, and thus, the obtained bearing capacity must be treated as the upper limit of the true limit load. The proposed approach is limited to bearing capacity estimations and is not dedicated to serviceability limit states, e.g., Johari et al. [30]. In the performed analyses, seismic loadings are not examined, and thus, the obtained results are reliable for a static scenario (this can influence the obtained results, e.g., [31]). However, this type of analyses will be explored in future research.

Based on the results presented in section 5 and the description of the proposed algorithm given in section 2 and section 3, the following conclusions can be drawn:

1. The algorithm proposed in this study demonstrates that the method of joining Vanmarcke’s spatial averaging with kinematic failure mechanisms can deliver an efficient approach for analysing two-layered soil problems if the bottom layer is spatially variable. Further efficiency improvement is possible if a constant covariance matrix is applied [32].
2. The obtained results indicate that the observed reduction in the bearing capacity coefficient of variation (see Figs 6 and 7) is caused mostly by three factors: thickness of the homogenous sand layer, length of the averaging domain *a*, and fluctuation scales that characterize the spatial variability of the undrained shear strength within the bottom layer. The impact of surcharge load is not considered in the performed analyses. The soft layer is assumed to be weightless (see arguments given in section 2.1); however, in future studies, it would not be very difficult to extend the analyses by soil weight consideration.
3. Three-dimensional issues are of special importance when soil spatial variability is taken into account in the numerical analyses. Two-dimensional simplifications provide a more conservative approach that can be significant in some scenarios, as shown in Figs 6 and 7.
4. As shown in the results section, the impact of dumping the feature of the thickness of the homogenous sand layer can be separated from the other impacts detailed in the second conclusion. An example of this separation is shown in Fig 9, where the reduction in the bearing capacity coefficient of variation is caused only by the homogenous sand layer. As expected, the reduction is greater for a greater sand thickness. This simple approach can be utilized in practical applications for creating graphs that allow nearly instant determination of the reduction in the bearing capacity coefficient of variation.

## Appendix A

The algorithm for computing average undrained shear strengths in the case of 11 rigid block failure mechanisms.

Step 1. Find the mean value $\mu_{Y,c_{u}}$ and the variance $\sigma_{Y,c_{u}}^{2}$ of the underlying normal distribution based on $\mu_{c_{u}}$ and $\sigma_{c_{u}}$ from step I (see section 3).
  $$\sigma_{Y,c_{u}}^{2}=ln\left(1+\frac{\sigma_{c_{u}}^{2}}{\mu_{c_{u}}^{2}}\right)$$ (A.1)
  $$\mu_{Y,c_{u}}=\text{ln}\;\left(\mu_{c_{u}}\right)-\frac{1}{2}{\sigma_{Y,c_{u}}}^{2}$$ (A.2)
Step 2. Take 16 values of undrained shear strength generated in step I in section 3 as a vector of stochastically independent components $Y_{c_{u}}=(c_{Y,u,1},\ldots ,c_{Y,u,16})$.
Step 3. Transform vector $Y_{c_{u}}$ to the lognormal distribution $X_{c_{u}},X_{c_{u},i}=exp\left(Y_{c_{u},i}\right)$, where *i* = 1, …, 16.
Step 4. Determine the covariance matrix [*C*_*X*_] (see Eq (13)) based on the optimal failure geometry established in step II in section 3.
Step 5. Compute the correlation coefficients for the covariance matrix determined in step 4.
  $${[r}_{X}(i,j)]=\frac{{[C}_{X}(i,j)]}{\sqrt{{[C}_{X}\left(i,i\right){][C}_{X}(j,j)]}},$$ (A.3)
  where *i*, *j* = 1, …, 16.
Step. 6. Transform [*r*_*X*_] to the corresponding normal underlying distribution correlation matrix [*r*_*Y*_], (see [7]):
  $${[r}_{Y}(i,j)]=\frac{ln\left(1+{[r}_{X}(i,j)]v_{X_{i}}v_{X_{j}}\right)}{\sqrt{\text{ln}\;\left(1+{v_{X_{i}}}^{2}\right)\text{ln}\;\left(1+{v_{X_{j}}}^{2}\right)}},$$ (A.4)
  where
  $$v_{X_{i}}=\frac{\sqrt{{[C}_{X}(i,i)]}}{\mu_{X_{c_{u}}}}$$ (A.5)
Step 7. Transform [*r*_*Y*_] to the corresponding normal underlying distribution covariance matrix [*C*_*Y*_],
  $${[C}_{Y}(i,j)]={[r}_{Y}(i,j)]\sqrt{Var\left(Y_{i}\right)Var(Y_{j})}$$ (A.6)
  where
  $$Var\left(Y_{i}\right)=ln\left(1+\frac{{[C}_{X}(i,i)]}{\mu_{X_{c_{u}}}^{2}}\right)$$ (A.7)
  where *i*, *j* = 1, …, 16.
Step. 8. Calculate the Cholesky decomposition of [*C*_*Y*_]:
  $${[C}_{Y}]=\left[L\right][{L]}^{T},$$ (A.8)
  where [*L*] is the lower triangular matrix. This matrix has real and positive diagonal entries (see [25]).
Step. 9. Standardize vector $Y_{c_{u}}$ obtained in step I in section 3.
  $$Y_{c_{u},i}^{*}=\frac{Y_{c_{u},i}-\mu_{Y_{c_{u}}}}{\sigma_{Y_{c_{u}}}}$$ (A.9)
  where *i*, *j* = 1, …, 16.
Step 10. Compute vector $Z_{c_{u}}$ of correlated entries by using the following theorem (see [17]): if [*C*_*Y*_] is a positively definite *n* × *n* matrix, $Y_{c_{u}}^{*}$ is an *n*-dimensional vector whose components are independent Gaussian standard random variables, and [*L*] is a lower triangular matrix such that Eq (A.8) is true, then, the random vector defined as $Z_{c_{u}}=[L]{[Y}_{c_{u}}^{*}]$ is the Gaussian vector with the covariance matrix [*C*_*Y*_]. As a result of using this theorem, entries of the vector $Z_{c_{u}}$ have expected values equal to zero and the covariance matrix [*C*_*Y*_]. To shift the components of $Z_{c_{u}}$ to proper mean values, they are added to $Z_{c_{u}}:P_{c_{u},i}=Z_{c_{u},i}+\mu_{Y_{c_{u},i}}$, where *i* = 1, …, 16.
Step 11. Transform $P_{c_{u}}$ to lognormal distribution:
  $$\bar{T_{c_{u},i}}=\text{exp}\left(P_{c_{u},i}\right)$$ (A.10)
  where *i* = 1, …, 16.
  The obtained components of $\overset{-}{T_{c_{u}}}=(\overset{-}{c_{u,1}},\ldots ,\overset{-}{c_{u,16}})$ are used in step IV in the algorithm given in section 3.
